# Renal function evaluation in patients with American Cutaneous Leishmaniasis after specific treatment with pentavalent antimonial

**DOI:** 10.1186/1471-2369-13-44

**Published:** 2012-06-20

**Authors:** Rodrigo A Oliveira, Cláudio G Lima, Rosa Ms Mota, Alice Mc Martins, Talita R Sanches, Antônio C Seguro, Lúcia C Andrade, Geraldo B Silva Junior, Alexandre B Libório, Elizabeth F Daher

**Affiliations:** 1 Department of Internal Medicine, School of Medicine, Federal University of Ceará, Campus Cariri, Rua Divino Salvador, 284, Centro, Barbalha, Ceará, Brazil, CEP-63180-000; 2 Department of Statistics, Sciences Center, Federal University of Ceará, Fortaleza, Ceará, Brazil; 3 School of Pharmacy, Federal University of Ceará, Fortaleza, Ceará, Brazil; 4 Division of Nephrology, School of Medicine, University of São Paulo, São Paulo, Brazil; 5 Department of Internal Medicine, School of Medicine, Federal University of Ceará, Campus Fortaleza, Fortaleza, Ceará, Brazil; 6 School of Medicine, Health Sciences Center, University of Fortaleza, Fortaleza, Ceará, Brazil; 7 Brazilian National Research Council (CNPq), São Paulo, Brazil

**Keywords:** American cutaneous leishmaniasis, Renal function, Tubular defects, Treatment

## Abstract

**Background:**

Renal evaluation studies are rare in American Cutaneous Leishmaniasis (ACL). The aim of this study is to investigate whether specific treatment reverts ACL-associated renal dysfunction.

**Methods:**

A prospective study was conducted with 37 patients with ACL. Urinary concentrating and acidification ability was assessed before and after treatment with pentavalent antimonial.

**Results:**

The patients mean age was 35.6 ± 12 years and 19 were male. Before treatment, urinary concentrating defect (U/P_osm_ <2.8) was identified in 27 patients (77%) and urinary acidification defect in 17 patients (46%). No significant glomerular dysfunction was observed before and after specific ACL treatment. There was no reversion of urinary concentrating defects, being observed in 77% of the patients before and in 88% after treatment (p = 0.344). Urinary acidification defect was corrected in 9 patients after treatment, reducing its prevalence from 40% before to only 16% after treament, (p = 0.012). Microalbuminuria higher than 30 mg/g was found in 35% of patients before treatment and in only 8% after treatment. Regarding fractional excretion of sodium, potassium, calcium, phosphorus and magnesium, there was no significant difference between pre and post-treatment period.

**Conclusion:**

As previously described, urinary concentrating and acidification defects were found in an important number of patients with ACL. Present results demonstrate that only some patients recover urinary acidification capacity, while no one returned to normal urinary concentration capacity.

## Background

Leishmaniasis is a parasitic disease caused by *Leishmania* species which could manifest as visceral, mucous or cutaneous involvement, depending on the host immune response [1-4].

It is estimated that 1.5 to 2 million people develop symptomatic disease each year [5]. American Cutaneous Leishmaniasis (ACL) is endemic in some countries in Africa, Middle East, Europe and Latin America, representing a Public Health problem, not only due to its high incidence, but also due to its potential to cause destructive and incapacitating lesions, with high psychosocial impact [4,6].

Renal involvement has been described in visceral leishmaniasis (kala-azar), including from mild urinary abnormalities to severe glomerular involvement and renal failure [7-10]. Renal failure in kala-azar is thought to occur due to interstitial nephritis secondary to immune complex deposition and hypersensitivity to pentavalent antimonials [6,11-13]. Tubular dysfunction has also been described in kala-azar as urinary concentrating and acidification defects, with few clinical manifestations [14].

In ACL there are very few studies regarding renal function evaluation, and the renal abnormalities described are linked to specific treatment with pentavalent antimonials [15]. These drugs are described to cause acute kidney injury due to hypersensitivity reaction [12]. Other possible mechanisms for kidney injury in ACL have not been investigated by now.

A recent study conducted by our study group found important abnormalities in ACL. Expression of the Na^+^/H^+^ exchanger (NHE3), H^+^-ATPase, and pendrin were all significantly higher in patients with ACL when compared with normal subjects. A combined urinary concentration and acidification defect was found in 32.4% of patients, before specific ACL treatment [16].

The aim of this study is to investigate if these tubular abnormalities persist after specific ACL-treatment.

## Methods

### Patients

This is a prospective study with 37 patients with confirmed diagnosis of ACL (epidemiologic, clinical and laboratorial) in a public health service in the city of Barbalha, Ceara, Brazil, between July 2008 and July 2009. Exclusion criteria was patients under 15 years-old or older than 60 years, use of pentavalent antimonials in the last 30 days, hypertension (Systolic blood pressure ≥ 140 or Diastolic blood pressure ≥ 90 mmHg), diabetes mellitus, urinary tract infection, systemic lupus erythematosus and other colagenosis, and previous kidney disease. The 37 patients were compared with 10 healthy volunteers. The protocol of this study was revised and approved by the Ethical Comitee of the Walter Cantidio University Hospital, Federal University of Ceara, Fortaleza, Brazil. Patients were included in the study after signing the informed consent form.

### Diagnosis of ACL

Diagnosis of ACL was based on epidemiologic and clinical criteria, Montenegro skin test and identification of parasite in tissue biopsy.

### Clinical and laboratory parameters

At the time of medical consult all symptoms and signals were evaluated, as well as race, age, gender, previous chronic diseases, number of skin lesions, time of disease, use of drugs, body mass index, blood pressure, Montenegro skin test, ACL classification. The following laboratory tests were studied in blood and plasma: urea (P_Ur_), creatinine (P_Cr_),pH, (P_osm_) osmolality, bicarbonate (BIC_s_), sodium (P_Na+_), potassium (P_K+_), chloride (P_Cl-_), magnesium (P_Mg++_), calcium (P_Ca++_), phosphorus (P_P-_), albumin/globulin, amilase, fast glucose, erythrocyte sedimentation rate (ESR). In urine were studied the following tests: creatinine and urea (U_Cr_ e U_Ur_), sodium (U_Na+_), potassium (U_K+_), chloride (U_Cl-_), calcium (U_Ca++_), phosphorus (U_P-_), magnesium (U_Mg++_), microalbuminuria, urinalysis, osmolality (U_osm_) and pH (U_pH_).

### Renal function evaluation

Glomerular filtration rate (GFR) was estimated through the Cockroft & Gault formula and it was considered abnormal when ≤ 90 ml/min/1,73 Body Surface Area m^2^. In an isolated urine sample collected before concentration and acidification tests, pre- and post-glucantime treatment sodium, potassium, chloride, magnesium and microalbuminuria were measured.

All patients underwent food and water deprivation for 12 hours. Fraction excretion of sodium (FE_Na_), potassium (FE_k_), calcium (FE_Ca_), phosphorus (FE_P_), magnesium (FE_Mg_) were calculated by standard formula. Microalbuminuria was measured in an isolated urine sample and normalized by urinary creatinine.

Urinary concentration ability was evaluated through the ratio between urinary and serum osmolality (U/P_osm_) after 12 hours water deprivation, and urinary osmolality (U_osm_) was measured before and 4 hours after administration of intranasal DDAVP® (desmopressin acetate 20mcg/kg - T_0_ and T_4_). Urinary acidification was evaluated by the urinary pH before and after administration of oral CaCl_2_ 2 mEq/kg (T_0_ and T_4_). Acidification defect was determined by the inability in decreasing U_pH_ for less than 5.50.

All patients underwent standard treatment with antimonial (Glucantime®) in the dose of 20 mg/kg/day for 20 days. All tests were done before treatment and 4 weeks after the beginning of the treatment.

### Groups definition

Pre-glucantime group – Tests performed before the beginning of treatment.

Post-glucantime group – Tests performed 4 weeks after the beginning of the treatment.

### Analytical methods

Urea: Determined by colorimetric uricase method (Labtest^®^). The results were expressed in mg/dl. Serum and urinary creatinine: Determined by colorimetric methods, picric acid, Taussky and Bonsness (Labtest^®^). The results were expressed in mg/dl. Serum and urinary Sodium and potassium (P_Na+_ e P_K+_): Determined by photometry technique with spectrophotometry, model B462 MICRONAL (Instrumentation Laboratory, Inc. USA). The results were expressed in mEq/L. Albumin: Determined by bromocresol reaction (Labtest^®^). The results were expressed in g/dl. Globulin: Determined by bromocresol reaction (Labtest^®^). The results were expressed in g/dl. Glucose: Determined by colorimetric glucose oxidase method (Labtest^®^). The results were expressed in mg/dl. Alkaline phosphatase: Determined by phosphatase kinetic method, Bowers and Mc Comb modified (Labtest^®^). The results were expressed in U/L. Amilase: Determined by colorimetric Caraway modified method. Results expressed in U/dl. pH, bicarbonate (HCO3^-^): were determined through “Blood gas analyser” machine (chiron diagnostic 238 - Bayer^®^). The results were expressed in mEq/L for bicarbonate. Urinary pH (U_pH_): measured by pHmetro Digital pG1000, model GEHAKALT. Urinary osmolarity: Determined by the technique pressure steam in osmometer model 5100 C (Wescor Inc., USA). The results were expressed in mOsm/Kg.H_2_O. Microalbuminuria: measured through immunoturbidimetry methods, using Tina-quant^®^ kit (Roche) and the results were expressed in mcg/g creatinine.

### Statistical analysis

All quantitative data are expressed as mean ± SEM. Differences between two parameters were analyzed either by paired Student t test or by nonparametric methods (Wilcoxon test and Mann–Whitney test). Chi-square test was used to analyze categoricalvariables. Values of P < 0.05 were considered statistically significant.

## Results

Of the 59 patients enrolled in the study with previously diagnosed of ACL on the basis of epidemiological, clinical, biochemical, and histopathological findings 22 were excluded: eight for testing negative on a new histopathological exam; nine for being under 15 years of age or over 60 years of age; two for subsequently declining to participate in the study; one for having hypertension; one for having diabetes mellitus; and one for having used an antimonial (meglumine antimoniate) within the last 30 days. Therefore, the study group included 37 ACL patients who agree to participate. The mean age was 35.6 ± 12 years and 19 (51.4%) were male. Clinical and demographic data were similar between ACL patients and controls (Table 1).

**Table 1:** Clinical and demographic data of patients with ACL compared with healthy subjects

**Characteristics**	**ACL (n = 37)**	**Control (n = 10)**	** *P* **
**Age (years)**	35.6 ± 12	32.3 ± 11.7	0.442
**Gender**			
Male	19 (51.4%)	6 (60%)	0.73
Female	18 (48.6%)	4 (40%)	
**Time of disease (days)**	28.5 ± 20.6	-	-
**Montenegro skin test (+/−)**	22/37	-	-
**Number of skin lesions**			
1	27 (72.9%)	-	-
2 to 4	7 (18.9%)	-	-
>4	3 (8.1%)		
**Systolic blood pressure, mmHg**	122 ±10	117 ± 9.5	0.221
**Diastolic blood pressure, mmHg**	80 ± 4.7	75 ± 8.5	0.079

Data expressed as mean ± standard deviation or %. Student t test.

Montenegro skin test was positive in 59.5% of cases. All patients have ACL in its isolated cutaneous type, 27 had a solitary skin lesion, 7 had 2–4 lesions and 3 had more than 4 lesions. The mean time of disease was 28.5 ± 20.6 days (range 7–90 days).

The laboratory evaluation, before and after treatment with pentavalent antimonial, is shown in Table 2. It was observed only a mild decrease in hemoglobin post-treatment. In biochemical analysis it was noted an increase in aminotransferases (p < 0.05) after treatment. No one patient presented increase in serum amylase with treatment.

**Table 2:** Laboratory data of 37 patients with ACL before and after specific treatment

	**Pre-treatment**	**Post-treatment**	** *P* **
**Hematocrit (%)**	42.6 ± 4.1	40.5 ± 4.2	0.0052
**Hemoglobin (g/%)**	13.9 ± 1.2	13.3 ± 1.5	0.019
**White blood count (/mm**^**3**^**)**	6.359 ± 1918	6.035 ± 1513	0.269
**Platelets (/mm**^**3**^**)**	275.351 ± 66.259	292.702 ± 60.570	0.074
**Arterial pH**	7.35 ± 0.1	7.35 ± 0.1	0.634
**Fasting glucose (mg/dl)**	80 ± 15	80 ± 17	0.915
**Amylase (U/dl)**	143 ± 48	148 ± 62	0.666
**Total Bilirubin (mg/dl)**	0.5 ± 0.27	0.6 ± 0.23	0.810
**Alkaline Phosphatase (U/L)**	93 ± 33	107 ± 52	0.08
**Albumin (g/dl)**	4 ± 0.6	4 ± 0.6	0.696
**Globulin (g/dl)**	3.3 ± 0.86	3.3 ± 0.7	0.135
**AST (g/dl)**	34 ± 15	46 ± 36	0.034
**ALT (U/L)**	28 ± 19	42 ± 33	0.023
**ESR (mm/h)**	30 ± 21	26 ± 18	0.198

Significant *P* < 0.05. AST: aspartate aminotransferase; ALT: alanine aminotransferase; ESR: erythrocyte sedimentation rate. Data expressed as mean ± standard deviation or %. T test and Wilcoxon test.

There was no significant glomerular filtration abnormality before and after treatment (109.6 ± 32 vs. 109.6 ± 28 ml/min/1.73 m^2^, *P* = 0.694). Microalbuminuria was 23.6 ± 26 mg/g creatinine, before treatment, and 14.6 ± 18.9 mg/g creatinine after treatment (p = 0.02). Urinary concentrating defect, based on U/Posm (<2,8), was observed in 27 patients before treatment and in 30 after treatment (77% vs. 88%, p = 0,344), with no significant difference between pre and post-treatment (2.2 ± 0.7 vs. 1.9 ± 0.75, *P* = 0.718) (Table 3).

**Table 3:** Glomerular and tubular function of 37 patients with ACL before and after specific treatment

	**Pre-glucantime (N = 37)**	**Post-glucantime (N = 37)**	**Control (N = 10)**
**P**_**creat**_**, (mg/dl)**	0.81 ± 0.16	0.81 ± 0.15	0.85 ± 0.18
**CrCl (ml/min/1.73 m**^**2**^**)**	109.6 ± 31.5	108.4 ± 28.5	116.4 ± 22.7
**U/P**_**osm**_**T4**	2.19 ± 0.73	1.95 ± 0.73	3.47 ± 0.33
**U**_**pH**_**T4**	5.45 ± 0.64	5.19 ± 0.60^*^	4.82 ± 0.20 ^#^
**U**_**osm**_**T4**	618 ± 202	552 ± 210	965 ± 81 ^#^
**FENa, (%)**	1.15 ± 0.74	1.35 ± 1.51**	0.73 ± 0.39
**FEk, (%)**	10 ± 6.6	10.1 ± 7.6	7.50 ± 2.8^#^
**FECa (%)**	1.07 ± 0.72	1.32 ± 1.01	0.62 ± 0.34^#^
**FEPO4 (%)**	10.9 ± 9.98	10.9 ± 15.7	9.10 ± 6.4
**FEMg (%)**	1.81 ± 1.70	1.90 ± 1.44	0.90 ± 0.40^#^
**Microalbuminuria (mg/g creatinine)**	23.6 ± 26***	14.6 ± 18.9	6.12 ± 4.06

* Pre vs. Post-glucantime, p = 0.0066; # control vs. Pre and post-glucantime, p < 0.05; **Post-glucantime vs. control, p = 0.048; ***pre vs. post-glucantime, p = 0.025. DATA: MEAN ± SD. Student t test, Mann–Whitney.

P_crea_ – SerumCreatinine.

CrCl – Creatinine clearance.

U/P_osm_ – Urinary and serum osmolarity ratio.

U_pH_ em T4 – Urinary pH in T4.

U_osm_ T4 – Urinary osmolality in T4.

FE: fraction excretion.

Urinary acidification defect, defined as the inability to reduce urinary pH to < 5.5 after CaCl_2_ administration, was observed in 15 patients before treatment and in only 6 after treatment (40% vs 16%, p = 0.012), with significant difference when comparing the pH values before and after treatment (5.50 ± 0.64 vs. 5.19 ± 0.60, *P* = 0.0066). After treatment, 18 among 36 patients (50%) presented P_HCO3_- <21 mEq/L, and pH <7.35 was seen in 42% cases. Regarding excretion fractions (FENa, FEk, FECa, FEP and FEMg) there was no significant differences in the values before and after treatment (Table 4). FE_Na+_ > 2% was found in 4 patients (10.8%), FE_k+_ > 10% in 11 patients (29.7%), FE_Ca++_ > 3% in 2 patients (5.4%), FE_PO4_- > 10% in 10 patients (29%) and FE_Mg++_ > 6% in only 1 patient (2.7%).

**Table 4:** Prevalence of renal dysfunction in 37 patients with ACL before and after specific treatment

	**Pre-treatment**	**Post-treatment**	**P**
**P**_**creat**_**>1.2 mg/dl**	-	-	-
**CrCl <90 ml/min/1.73 m**^**2**^	11 (30%)	12 (32%)	1.000
**Microalbuminuria > 30 mg/g creat**	12 (39%)	3 (10%)	0.004
**U**_**osm**_**T4 < 700mmOsm/kg H**_**2**_**O**	21 (62%)	24 (71%)	0.508
**U/P**_**osm**_**T4 < 2.8**	26 (77%)	30 (88%)	0.344
**U**_**pH**_**T4 > 5.5**	15 (40%)	6 (16%)	0.012
**EF**_**Na+**_ **> 2%**	5 (14%)	4 (11%)	1.000
**EF**_**k+**_ **> 10%**	12 (32%)	11(30%)	1.000
**EF**_**Ca++**_ **> 3%**	2 (5%)	2 ( 5%)	1.000
**EF**_**PO4-**_ **> 10%**	17 (46%)	10 (27%)	0.143
**EF**_**Mg++**_ **> 6%**	1 (2.7%)	1 (2.7%)	1.000

P_crea_ – plasma creatinine; Cr_Cl_ – creatinine clearance; U/P_osm_ – urine and plasma osmolarity ratio; U_pH_ T4 – urine pH in T4 (4 hours after CaCl_2_ administration); U_osm_ T4 – urine osmolariry in T4 (4 hours after DDAVP administration); EF_Na_ – Sodium excretion fraction; EF_K_ – Potassium excretion fraction; EF_Ca_ – Calcium excretion fraction; EF_Mg_ – Magnesium excretion fraction. Data expressed as percentage (%).

None of the patients presented severe adverse reactions to the pentavalent antimonial. Among the 37 studied cases, 5 (13.5%) presented low degree fever, myalgia and asthenia, and one patient had arthralgia and headache.

## Discussion

The results of the present study evidence the occurrence of asymptomatic tubular dysfunction probably induced by ACL and that partially improved after specific treatment.

All patients studied had ulcerated lesions. They were in very early stages of the disease, however they presented systemic inflammatory reactions, such as fever, arthralgias and myalgias, which have been previously reported [17]. This can lead to humoral activation, which can explain the finding of renal tubular dysfunction.

In the present study, the number of patients with Cl_Cr_ < 90 ml/min/1.73 m^2^ did not present significant difference before and after treatment with pentavalent antimonial (11 among 37 before treatment and 12 among 37 after treatment). Previous reports on the renal involvement in ACL have linked renal abnormalities to the use of these drugs, as acute kidney injury by tubulointerstitial nephritis or reaction Jarish-Herxheimer like, which is not in accordance to our findings [12,15].

Renal dysfunction in visceral leishmaniasis (kala-azar) has been described [7-10,18]. Lima Verde et al [14] in a study with 50 patients with kala-azar found GFR < 80 ml/min/1.73 m^2^ in 14 cases (28%). In another study, including 224 patients with kala-azar, acute kidney injury was found in 76 cases (33.9%) and this complication was associated with increased mortality [10]. In a recent study by Daher et al [19], renal tubular dysfunction in kala-azar significantly improved after treatment with pentavalent antimonial. After specific treatment, all patients in the present study still remained with urinary concentrating and two-thirds improved previous acidification defects, suggesting that the tubular damage can be irreversible.

In our study microalbuminuria higher than 30 mg/g was found in 35% of patients before treatment and in only 8% after treatment, suggesting that glomerular lesion in ACL could have been caused by the parasitic disease *per se*, but without GFR reduction. In a study with 11 patients with kala-azar, 8 presented increased microalbuminuria (81.8%), which is higher than that found in ACL [7]. Elnojomi et al [20] detected abnormal microalbuminuria in 35 out of 88 (40%) patients with kala-azar, with no glomerular dysfunction. Microalbuminuria can also be found in other infectious diseases that affect the skin and nerves, such as leprosy. Oliveira et al [21] identified microalbuminuria in 4 out of 59 patients with multibacillary leprosy (8.5%). A higher prevalence of microalbuminuria was found in another study involving leprosy patients. Kirsztajn et al [22]. identified microalbuminuria higher than 20 mg/l in 15.8% of 96 patients with leprosy. In the present study we observed a decrease in microalbuminuria after treatment, which suggests that ACL per se can lead to increased urinary albumin loss. However microalbuminuria is not yet a well defined marker of glomerular dysfunction in infectious diseases.

In the present study, the urinary concentrating ability was evaluated by the ratio U/P_osm_ and U_osm_, measured after 12 h water deprivation and fasting, which was sensitized by the administration of DDAVP. Urinary concentration deficit was found in 27 cases (77%) before treatment and in 31 (88%) after treatment (p = 0.344). There was also no significant difference in the values of U_osm_ after the administration of DDAVP before and after treatment (p = 0.508). Based on these findings we can suggest that ACL *per se* can cause urinary concentrating deficit. The specific treatment did not improve this abnormality, but do not allow a worsening in the tubular lesion.

In a study involving 11 patients with ACL treated with pentavalent antimonial, 40 mg/kg for 30 days, the persisting urinary concentrating deficit was observed in 8 cases (72.7%) [23]. Veiga et al [24], reported 5 cases of ACL treated with pentavalent antimonial in conventional doses, but with a longer duration, which developed urinary concentrating inability. Lima Verde et al [14], found 68% of urinary concentrating capacity defect in patients with kala-azar before pentavalent antimonial therapy. The persistence of this abnormality can be a consequence of ACL itself, which can cause a severe tubular damage. Further investigations, with a longer period of observation after ACL treatment, would be important to establish the long-term outcomes regarding this complication. Maybe urinary concentrating ability can be restored with time in this group of patients.

In the present study urinary acidification defect was found in 40% of the patients before treatment and in 16% after treatment, which suggests an important improvement in acidification ability after specific treatment for ACL. Urinary acidification deficit is less common than concentration deficit. In a study with patients with kala-azar, urinary acidification defect was found in 64% of cases after specific treatment [14].

## Conclusions

Renal abnormalities detected in ACL improve partially after specific treatment, especially microalbuminuria and urinary acidification. The persistent urinary concentranting deficit may be related to a permanent damage induced by ACL or maybe an overlap effect of the treatment and the disease. Further studies are required to better understand the mechanisms involved in tubular dysfunction caused by ACL and by the treatment.

## Competing interests

The authors declare that they have no competing interests.

## Authors' contributions

RAO and CGL carried the patients`evaluation and collection of biological samples for laboratory tests and drafted the manuscript. TRS and AMCM carried out the laboratory tests. RMSM participated in the design of the study and performed the statistical analysis. RAO, ACS, LCA, GBSJ, ABL and EFD conceived the study, and participated in its design and coordination and helped to draft the manuscript. All authors read and approved the final manuscript.

## Pre-publication history

The pre-publication history for this paper can be accessed here:

http://www.biomedcentral.com/1471-2369/13/44/prepub
